# Brain dynamics that correlate with effects of learning on auditory distance perception

**DOI:** 10.3389/fnins.2014.00396

**Published:** 2014-12-09

**Authors:** Matthew G. Wisniewski, Eduardo Mercado, Barbara A. Church, Klaus Gramann, Scott Makeig

**Affiliations:** ^1^711th Human Performance Wing, U. S. Air Force Research LaboratoryWright-Patterson Air Force Base, OH, USA; ^2^Department of Psychology, University at Buffalo, The State University of New YorkBuffalo, NY, USA; ^3^Biological Psychology and Neuroergonomics, Berlin Institute of TechnologyBerlin, Germany; ^4^Swartz Center for Computational Neuroscience, Institute for Neural Computation, University of California, San DiegoSan Diego, CA, USA

**Keywords:** electroencephalography (EEG), perceptual learning, familiarity, independent component analysis (ICA), ranging, event-related spectral perturbation (ERSP)

## Abstract

Accuracy in auditory distance perception can improve with practice and varies for sounds differing in familiarity. Here, listeners were trained to judge the distances of English, Bengali, and backwards speech sources pre-recorded at near (2-m) and far (30-m) distances. Listeners' accuracy was tested before and after training. Improvements from pre-test to post-test were greater for forward speech, demonstrating a learning advantage for forward speech sounds. Independent component (IC) processes identified in electroencephalographic (EEG) data collected during pre- and post-testing revealed three clusters of ICs across subjects with stimulus-locked spectral perturbations related to learning and accuracy. One cluster exhibited a transient stimulus-locked increase in 4–8 Hz power (theta event-related synchronization; ERS) that was smaller after training and largest for backwards speech. For a left temporal cluster, 8–12 Hz decreases in power (alpha event-related desynchronization; ERD) were greatest for English speech and less prominent after training. In contrast, a cluster of IC processes centered at or near anterior portions of the medial frontal cortex showed learning-related enhancement of sustained increases in 10–16 Hz power (upper-alpha/low-beta ERS). The degree of this enhancement was positively correlated with the degree of behavioral improvements. Results suggest that neural dynamics in non-auditory cortical areas support distance judgments. Further, frontal cortical networks associated with attentional and/or working memory processes appear to play a role in perceptual learning for source distance.

## Introduction

Most of what is currently known about human auditory distance perception comes from research focused on variations in acoustic cues produced by propagation, and on how degrading or altering such cues affects distance judgments. This work has shown that listeners utilize intensity, spectral, binaural, and direct-to-reverberant energy features to judge distances and that judgments can be altered by interfering with feature perception (for review, see Zahorik et al., 2005; Fluitt et al., 2013). For example, changing the ambient properties of listening environments can make sources sound nearer or farther than they actually are (e.g., Mershon et al., 1989).

Interestingly, auditory distance perception also depends on experience, even between sounds with similar acoustic properties. Coleman (1962) had listeners judge distances of noise bursts and found that on the first experimental trial judgments were unreliable. In later trials accuracy improved, presumably as participants learned to gauge the intensities of the sound source via feedback (Coleman, 1962). Blind individuals discriminate auditory source distance better than normally sighted individuals (Voss et al., 2004; Kolarik et al., 2013), possibly reflecting learning-induced cortical plasticity in areas normally devoted to vision (e.g., Gougoux et al., 2004; Voss et al., 2011). Also, the source distance of speech played forward is more accurately judged than the same speech played backwards (McGregor et al., 1985; Brungart and Scott, 2001; Banks et al., 2007; Wisniewski et al., 2012). Because the known acoustic cues to distance are well matched between stimuli played forward and time-reversed (McGregor et al., 1985; Brungart and Scott, 2001), better performance for forward speech suggests that central cognitive processes play a significant role in distance perception. Important to note is that in all the above studies acoustic distance cues were identical or quite similar across conditions, demonstrating that auditory distance perception cannot be fully understood by focusing solely on the impact of cue alteration and degradation on distance judgments.

Cognitive neuroscience has advanced our understanding of the mechanisms involved in the azimuthal localization of sounds (e.g., Zatorre et al., 2002; Salminen et al., 2010) and may potentially be able to play a similar role in understanding the processes involved in auditory distance perception and effects of learning. However, research on the neural bases of distance perception has been limited. Some studies suggest a reliance on right lateralized auditory areas when intensity is a useful distance cue (Mathiak et al., 2003; Altmann et al., 2013). The left posterior superior temporal gyrus and planum temporale may be important for processing intensity independent cues, at least when sounds are presented on the right side of the inter-aural axis (Kopčo et al., 2012). There is also some evidence that judging distance of ecologically relevant sound sources involves cortical networks outside of traditional auditory areas. For instance, Seifritz et al. (2002) found that processing rising intensity (approaching or looming sources) compared to falling intensity sounds led to greater blood oxygen level-dependent (BOLD) signals in right parietal, motor, and pre-motor areas, in addition to left and right superior temporal sulci and middle temporal gyri.

We recently observed a distributed cortical network involved in auditory distance perception using electroencephalography (EEG). In that study, participants were tested on their ability to discriminate the distances of intensity normalized English and Bengali forwards and backwards speech prerecorded at near (2 m) and far (30 m) distances (Wisniewski et al., 2012). Replicating previous behavioral results, accuracy was higher for forward than backwards speech. Independent component analysis (ICA), a blind source separation method that finds spatially fixed and temporally independent component (IC) processes in multichannel EEG data, identified several cortical sources of EEG (cf. Makeig et al., 2004). Clusters of IC processes localized to a range of cortical areas including the medial frontal cortex, left and right superior temporal gyri, and parietal areas, showed significant event-related changes in oscillatory dynamics associated with making distance judgments.

There were also quantitative differences related to processing distance cues from different types of speech. For the left temporal IC process cluster, English speech trials showed the strongest event-related desynchronization (ERD) of the alpha rhythm (i.e., decreases in 8–12 Hz power). As alpha ERD can be considered to reflect a break from resting-state neural synchrony (for review see Pfurtscheller and Lopes da Silva, 1999) and/or a state of cortical excitation (Weisz et al., 2011), ERD in the left temporal cluster of ICs may have reflected the use of left-lateralized cortical speech areas for processing familiar speech (Boatman, 2004; Hickok and Poeppel, 2007) or enhanced processing of intensity independent distance cues (Kopčo et al., 2012). For IC processes localized at or near medial frontal cortex, event-related power increases (Event-related synchronization; ERS) in the high-alpha/low-beta range (10–16 Hz) were largest for accurately judged Bengali speech. In contrast, poorly perceived backwards speech samples induced relatively large transient ERS in the theta range (4–8 Hz) for a separate cluster of IC processes. ICs in this cluster showed scalp projections similar to scalp maps seen for late auditory-evoked potential (AEP) components (i.e., strong projection to central electrodes), suggesting that transient ERS was at least partially related to typically observed obligatory responses to auditory stimulation.

Medial frontal brain regions such as the anterior cingulate cortex have been implicated in sustained auditory attention (Paus et al., 1997; Zatorre et al., 1999; Benedict et al., 2002) and EEG work suggests that sources localized to nearby regions show sustained ERS that indexes cognitive demands placed on these frontal networks (Onton et al., 2005; Ahveninen et al., 2013). In contrast, transient ERS and concurrent ERP features have been linked to orienting (Barry et al., 2012), novelty processing (Debener et al., 2005), and auditory distraction (Schröger, 1996). That medial frontal source activities correlate with performance bolsters arguments that non-auditory brain regions have a significant role to play in auditory distance perception (Seifritz et al., 2002). Furthermore, the differences seen between speech categories in medial frontal, left temporal, and other possible AEP/ERP sources, suggest that analyses of larger scale brain dynamics are needed to understand the mechanisms driving learning-related effects. To date, little work has been done in this area. Learning-related effects have instead been attributed to “cognitive factors,” often with no attempt to explore what those factors may be or how they relate to processing in cortical areas outside of the canonical auditory system (Zahorik et al., 2005).

The current work builds on our earlier study by examining how training impacts accuracy and cortical processing across speech categories. A pre-/post-test design was employed wherein participants were initially tested on their distance perception accuracy, subsequently trained on the task, and then tested again. English, Bengali, and backwards samples of English and Bengali speech were used as stimuli. We expected that training would improve the accuracy of distance perception across speech categories and that the degree of improvement would be related to speech familiarity. It was also expected that the comparison of pre- and post-test EEG would clarify how the cortical networks described above are involved in auditory distance perception (Seifritz et al., 2002; Wisniewski et al., 2012). Specifically, we hypothesized that EEG dynamics previously associated with successful task performance (e.g., upper-alpha/low-beta ERS and alpha ERD) would be more evident in post-test than pre-test EEG. EEG features associated with speech categories showing poor distance perception accuracy should be less evident (e.g., large transient theta ERS for backwards speech). Although the current study was designed to measure how within-experiment learning interacts with speech familiarity, we also expected that the findings of the previous study, which focused exclusively on familiarity effects, would be replicated here. Specifically, we predicted that the quantitative differences in ERS/ERD patterns that we previously observed would be evident in the post-test. Although our main interest was in processes related to auditory distance perception learning, the study was not meant to identify features in EEG that are specialized for distance perception. EEG correlates of performance and learning in distance perception tasks may very well correlate with behavior in other non-distance-related and non-spatial listening tasks. A secondary goal of the study was thus to identify features in EEG that could potentially be explored in other, non-spatial tasks involving auditory perceptual learning. Most past studies of human auditory perceptual learning have focused on transient EEG features associated with AEPs rather than the time-frequency features we explored here.

## Materials and methods

### Ethics statement

The Human Research Protections Program of the University of California, San Diego approved the study. All participants signed an informed consent form before participating.

### Participants

The same 17 participants from our original study (Wisniewski et al., 2012) were paid to participate in additional training and post-test phases. All phases were run in a single session. Participants were fluent speakers of English with no fluency in Bengali. Two participants' data were dropped from analyses due to errors that occurred in data collection.

### Stimuli

One male speaker, fluent in English and Bengali, was recorded in the lab producing several English and Bengali phrases. Recordings were made on a Sony MD Walkman MZ-NH900 digital recorder (Sony Corporation of America, New York, NY) with an AKG D9000 microphone (frequency range: 20–20 kHz; AKG Acoustics, Austria). The microphone was placed ~15 cm from the speaker's mouth. Backwards speech tokens were created from a subset of English and Bengali phrases (italicized in Table 1) by reversing the speech waveforms. The selections of stimuli used for backwards speech tokens were based on previous behavioral work (McGregor et al., 1985; Brungart and Scott, 2001; Banks et al., 2007). English is the most familiar speech category as it is lexically and phonetically familiar to our sample of listeners. Bengali is less familiar than English due to no knowledge of word meaning, but is more familiar than backwards speech because of some phonetic content that overlaps with English (Barman, 2009).

**Table 1:** **Phrases recorded by a speaker that were later recorded at distances of 2 m and 30 m**.

**Speech sequence**	**Duration (ms)**
*Don't ask me to carry an oily rag like that*.	2544
*How far away do you think I am?*	1591
*Threat*.	277
*Warning*.	501
*Emergency*.	666
*Look out*.	500
Over here.	530
Caution.	561
Hello.	290
*Goodbye*.	520
Amaka ooghta tooltaa bolonah.	1734
*Aa kha nae*.	531
Aloo.	364
Kawla.	344
*Choo noo dau*.	632
Shaub dhan ah.	728
*Aamee kau tou dor ah ache*.	1589
Mo mosh Kar.	707
Hah.	408
Nah.	355

*Backwards stimuli were made from italicized phrases*.

All recordings were then broadcast from a SUNN speaker (Model 1201, Fender Musical Instruments Corporation, Scottsdale, AZ) in an open grass field at 2 m (near) or 30 m (far) away from the microphone using the same equipment as the original recordings. Recordings were made at night to minimize environmental noise. The mean amplitudes of recordings were normalized to ~−10 dB FS. The final stimulus set contained 20 tokens in each of the three speech categories (10 near, 10 far), yielding a total of 60 stimuli^1^.

**Figure 1:**
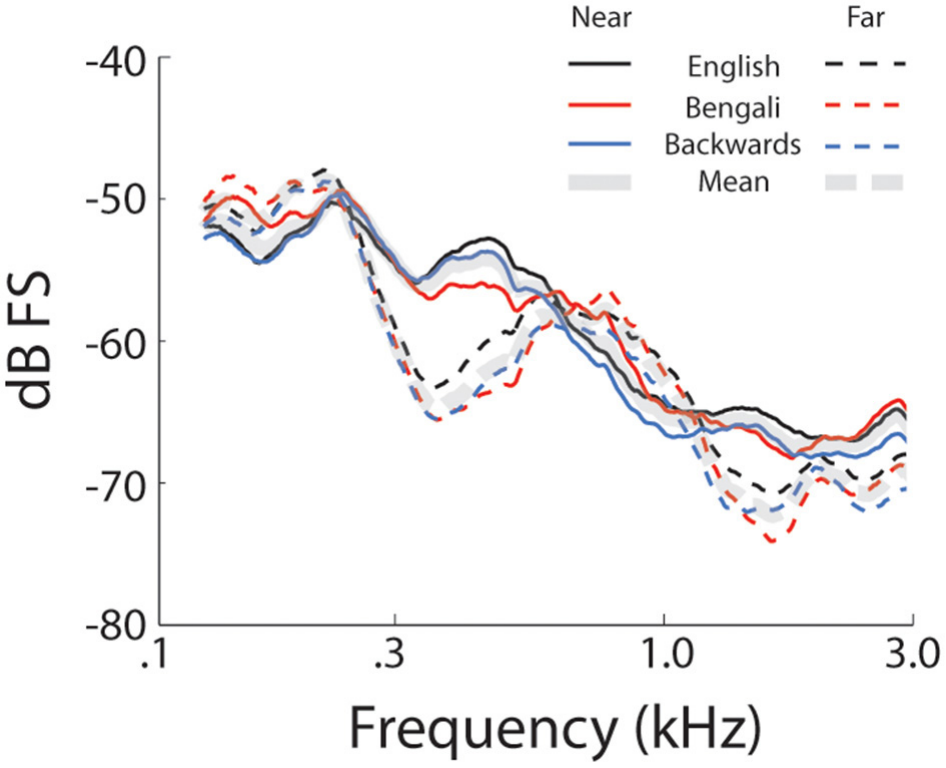
**Stimulus set spectra**. Mean spectra for each speech category at each level of distance. Solid lines represent near recordings and dashed lines represent far.

Figure 1 shows mean spectra for each speech category and distance. Spectra were analyzed in 5 single-octave bands (100–200 Hz, 200–400 Hz 400–800 Hz, 800–1600 Hz, and 1600–3200 Hz) to test for possible differences in cues to distance across speech categories in the stimulus set. A mixed-model 2 (Distance) × 3 (Speech Category) × 5 (Octave) ANOVA, treating Speech Category as a between subjects factor, found significant main effects of Distance [*F*_(1, 27)_ = 55.25, *p* < 0.001, η^2^_*p*_ = 0.67], and Octave [*F*_(4, 108)_ = 213.70, *p* < 0.001, η^2^_*p*_ = 0.88]. The main effect of distance trended such that power was lower for far sounds (i.e., the dashed lines in Figure 1 are on average lower than the solid lines). The main effect of octave relates to a drop in power with increasing frequency. There was also a significant Distance × Octave interaction [*F*_(4, 108)_ = 97.21, *p* < 0.001, η^2^_*p*_ = 0.78], possibly related, in part, to a greater rate of attenuation of higher frequencies with increasing distance (Zahorik et al., 2005). Another factor contributing to the interaction is differences in spectral peaks and notches. This difference between near and far distances may reflect decreased signal-to-noise ratio in recordings at far distances (cf. Zahorik et al., 2005). Because the amplitude normalization process increased the amplitude of far recordings, background noise was amplified along with speech signals, making it more evident in far recordings. The signal-to-noise ratio difference between near and far recordings, although amplified here, is an effective distance cue under more naturalistic conditions (Fluitt et al., 2013). No main effects or interactions with the Speech Category Factor were found (*p* > 0.55). The durations of stimuli (Table 1) are also similar across speech categories^2^. Overall, the stimulus set analysis shows that there are spectral cues to distance when overall amplitude cues are minimized, and that cue presence is comparable across speech categories. Although the current stimulus set does not contain all natural cues to distance perception (e.g., binaural cues), similar stimulus sets have proved informative for examining learning-related effects in distance perception (McGregor et al., 1985).

### Apparatus

Experimental procedures were executed using the ERICA software platform (Delorme et al., 2011) running on Windows XP. Stimuli were presented over computer speakers in a closed room placed ~1 m in front of subjects at a level not exceeding 75 dB SPL. Speakers were used to avoid interference from head or earphones in the placement of electrodes and collection of data from our high-density electrode array. Room and speaker arrangements were identical for each participant and did not change throughout the experiment. Any effects seen in behavior or EEG between conditions can therefore not be explained by differences in room acoustics. Listeners responded via a computer keyboard. Feedback was presented on a computer screen.

### Behavioral procedures

In a single-interval, two-alternative forced choice task (1i-2afc), participants were instructed to indicate whether a presented sound was near (2 m) or far (30 m) using only two fingers of their right hand, which were to remain on the keyboard near the “j” and “k” keys. Keys were labeled “N” (for near) and “F” (for far), respectively. Participants were made aware that the sounds were speech sounds recorded at different distances and then equalized in amplitude so that overall intensity was not a valid cue to distance.

There were three phases of the experiment: pre-test, training, and post-test. All phases employed the task described above and contained three blocks of 60 trials (one trial for each individual stimulus; see Table 1). The order of trials was randomized within a block. Feedback of correctness was presented during training. During the pre- and post-tests no feedback was provided.

### EEG acquisition and ICA decomposition

During the pre- and post-tests EEG was recorded from 248 channels at 24-bit A/D resolution, sampled at 512 Hz, and referenced to CMS-DRL of a Biosemi ActiveTwo system (Biosemi, Netherlands). A custom whole head electrode montage was used, the 3-D positions of which were recorded for each individual (Polhemus Inc, Colchester, VT). Water-based conductive gel was inserted into wells of the cap before placing electrodes in those wells. Voltage offsets for electrodes were brought within ± 20 μV, or were rejected from analysis when this criterion could not be met.

All offline analyses were conducted using the open source EEGLAB toolbox (Delorme and Makeig, 2004; http://sccn.ucsd.edu/eeglab) and custom scripts written in MATLAB (Mathworks, Natick, MA). Recorded EEG data was first re-sampled to 250 Hz, high-pass filtered (1 Hz), and then low-pass filtered (100 Hz). Channels containing excessive artifacts were rejected from analysis. Data segments containing high-amplitude, high-frequency muscle noise were rejected as well. Data was then re-referenced to the average voltage of the retained channels (134–224 channels per subject; *M* = 186, *SD* = 32).

EEG reflects a sum of brain and non-brain processes (e.g., muscle noise, eye movement artifact, line noise, etc.). To find maximally independent component processes in EEG, full-rank extended infomax ICA was applied to each individual's data using the *binica()* function in EEGLAB. Extended infomax ICA is a blind source separation algorithm that, under favorable circumstances, decomposes linearly mixed processes contributing to the EEG at scalp channels. An ICA decomposition of EEG data returns a spatially fixed and maximally temporally independent set of component processes without relying on *a priori* assumptions about the spatial distributions and temporal dynamics of those processes. The event-related dynamics of ICs can be analyzed with the same methods used to analyze event-related dynamics in channel data. For further information on the application of ICA in EEG research see Makeig et al. (1997, 2004).

ICs were fit with single equivalent current dipole models using each individual's recorded electrode locations fit to a template boundary element head model and then localized in the template brain using the *dipfit()* function in EEGLAB. ICs retained for later clustering (described below) were those for which the estimated equivalent current dipole was in the brain volume and for which the scalp projection of the equivalent dipole accounted for more than 85% of the variance in the IC scalp projection. An average of 19 ICs (*SD* = 7) were retained per participant. ICs identified as blinks, lateral eye-movements, or muscle-related artifacts were removed from channel data.

### Event-related spectral perturbations (ERSPs)

Following ICA decomposition, 4-s epochs (from 1 s before to 3 s after stimulus onsets) were extracted from the continuous data. A time-frequency approach to analysis was taken by examining ERSPs (Makeig, 1993). The *newtimef()* function of the EEGLAB toolbox was used to compute each IC's event-related spectrum using Morlet wavelets in a frequency range between 2 and 20 Hz (2 cycles at the lowest frequency to 10 cycles at the highest). Following this computation single trials were linearly time-warped to produce equal numbers of data points between stimulus onset and key presses in each trial. The mean spectrum from the pre-stimulus period (calculated using all epochs) was used as a divisive baseline (Gain model; see Grandchamp and Delorme, 2011) to determine relative power. The same method was used to compute ERSPs for channel data.

Single-trial time-frequency decompositions were also computed. For each trial a one-dimensional vector of spectral power within a specified frequency window was extracted from ERSPs (exact frequency bands given below). Then, all trials (combined across within-cluster ICs) were sorted by stimulus offset or response time (RT), smoothed over trials, and plotted. Both the time-warped ERSPs and single-trial sorting served to determine whether relative power within a time-frequency region of interest was related more strongly to stimulus processing (aligned to stimulus onset) or to stimulus offsets and key presses. For further detail on time-frequency decompositions and sorting see Supplementary Materials.

### AEPs

Although not critically related to our hypotheses, transient ERS is often associated with components of the AEP. Given that we expected to observe transient ERS, we computed AEP waveforms for Cz and its nearest neighboring 8 channels using data backprojected from: (1) all non-artifactual ICs and (2) the cluster of IC processes showing the clearest transient ERS. Channels surrounding Cz were selected on the basis of fronto-central scalp distributions for AEP components and the scalp projection of ICs within the cluster showing clear transient theta ERS at stimulus onset. Baseline correction used the 100 ms preceding the onset of stimuli. Waveform peaks were extracted by taking the maximal voltage deflections within the following time-windows: N1 (80–160 ms), P2 (160–260 ms).

### Cluster selection, statistics, and plotting

An automated K-means procedure was used to identify ICs within and across participants having similar scalp map topographies, equivalent current dipole locations, ERSPs, and mean log power spectra. A detailed description of clustering procedures is given in Supplementary Material.

Each IC's mean time-warped ERSP image was masked to reflect only significant perturbations from baseline (bootstrap resampling, *p* < 0.01). Displayed time-warped ERSPs represent an average of the masked individual ICs within an IC process cluster, masked further using a binomial test at each time-frequency point (*p* < 0.01; see Onton et al., 2005). To limit Type I error, we formally analyzed only IC clusters previously shown to be of interest and in time-frequency windows close to those in which we previously found differences between speech categories (Wisniewski et al., 2012). Analyses thus focused on a Central Midline cluster showing transient theta ERS (0.15–0.6 s; 4–8 Hz), a Frontal Midline cluster showing sustained upper-alpha/low-beta ERS (0.4–1.7 s; 10–16 Hz), and a Left Temporal cluster showing alpha ERD (0.5–2.45 s; 8–12 Hz). No attempt was made to optimize time-frequency windows. Pending determination of stimulus-related responses in time-warped and smoothed single-trial ERSPs, mean relative power measures within these windows were entered into 3 (Speech Category: English, Bengali, Backwards) × 2 (Test: Pre-test, Post-test) repeated measures ANOVAs.

To further characterize how changes in ERSPs from pre- to post-test related to perceptual learning for distance, Pearson correlations were calculated between behavioral improvement scores for each speech category and associated relative power changes. Both behavioral and EEG change measures were computed by subtracting pre-test from post-test measures. Some participants contributed more ICs per IC process cluster. When this was the case, the mean relative power change across an individual's ICs was entered into correlations.

We did not expect to see differences between near and far trials in ERSPs. Most studies reporting differences in electro/magnetic responses to acoustically similar stimuli employ oddball paradigms to get responses to some oddball stimulus that differ from a frequently presented one (for a distance-related study of this type, see Mathiak et al., 2003). We did not use such a task here because our goal was to characterize brain dynamics associated with processes of making distance judgments rather than to track responses related to representational differences along the dimension of distance (cf. Altmann et al., 2013; Kopčo et al., 2012; Mathiak et al., 2003). Nevertheless, we examined potential differences between near and far trials using 2 (Test: Pre-test, Post-test) × 2 (Distance: Near, Far) repeated measures ANOVAs. The factor of Distance was analyzed separately from Speech Category, because breaking up the analysis into all factors left a limited number of trials per condition.

When necessary for interpreting main effects or interactions, *post-hoc* paired-sample *t*-tests were conducted and interpreted with a false discovery rate (FDR) procedure (α = 0.05)^3^. Corrected *p*-values are reported. The same FDR procedure was used for interpreting correlations.

## Results

### Behavior

**Figure 2:**
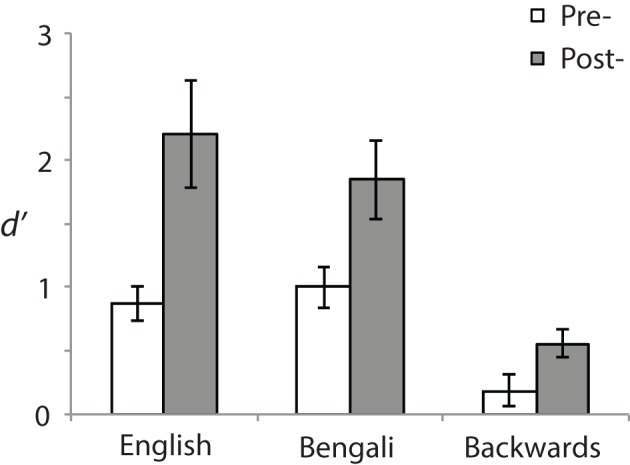
**Perceptual sensitivity (*d*′)**. Sensitivities to distance differences are shown for each speech category and test. Error bars show standard errors of means.

Behavior was analyzed using a signal detection measure for sensitivity according to the formula: *d*^′^ = *z* (*H*)− *z*(*F*). Correct responses to near stimuli were counted as “Hits” (H) and incorrect responses to far stimuli as false alarms (F) (see Macmillan and Creelman, 1991). Figure 2 shows *d*′ for each speech category at pre- (white bars) and post-test (gray bars). A 3 (Speech Category) × 2 (Test) ANOVA revealed a main effect of Speech Category [*F*_(2, 28)_ = 31.23, *p* < 0.001, η^2^_*p*_ = 0.69] stemming from differences in perceptual sensitivity. *Post-hoc* paired comparisons revealed that both English [*t*_(14)_ = 6.95, *p* < 0.001, *r*^2^ = 0.79] and Bengali [*t*_(14)_ = 6.38, *p* < 0.001, *r*^2^ = 0.76] were judged more accurately than backwards speech. Mean accuracies for English and Bengali speech were not significantly different (*p* > 0.47).

The main effect of Test [*F*_(1, 14)_ = 11.60, *p* = 0.004, η^2^_*p*_ = 0.45] and the Speech Category × Test interaction were also significant [*F*_(2, 28)_ = 5.10, *p* = 0.013, η^2^_*p*_ = 0.27]. Accuracy was greater in the post- than the pre-test for English [*t*_(14)_ = 3.06, *p* = 0.018, *r*^2^ = 0.40], Bengali [*t*_(14)_ = 3.20, *p* = 0.02, *r*^2^ = 0.42], and Backwards speech [*t*_(14)_ = 2.74, *p* = 0.028, *r*^2^ = 0.35]. Although learning related to perceptual sensitivity occurred for each speech category, the interaction suggests differential learning across speech categories. To further examine this, improvement scores were analyzed (Post-test minus Pre-test sensitivity). Mean improvements in *d*′ were as follows: English = 1.34 (*SE* = 44), Bengali = 0.85 (*SE* = 0.27), and backwards = 0.37 (*SE* = 0.13). Improvements were significantly greater for English [*t*_(14)_ = 2.47, *p* = 0.041, *r*^2^ = 0.30], and Bengali [*t*_(14)_ = 2.29, *p* = 0.049, *r*^2^ = 0.27], relative to backwards speech. The difference in learning between English and Bengali speech was not significant (*p* > 0.11).

In regards to distance judgment accuracy, behavioral data shows that: (1) sensitivity to differences in distance improved from pre- to post-test across speech categories; (2) English and Bengali speech were perceived more accurately than backwards speech; and (3) there was a learning advantage for English and Bengali over backwards speech^4^.

### Electrophysiology - channel data

**Figure 3:**
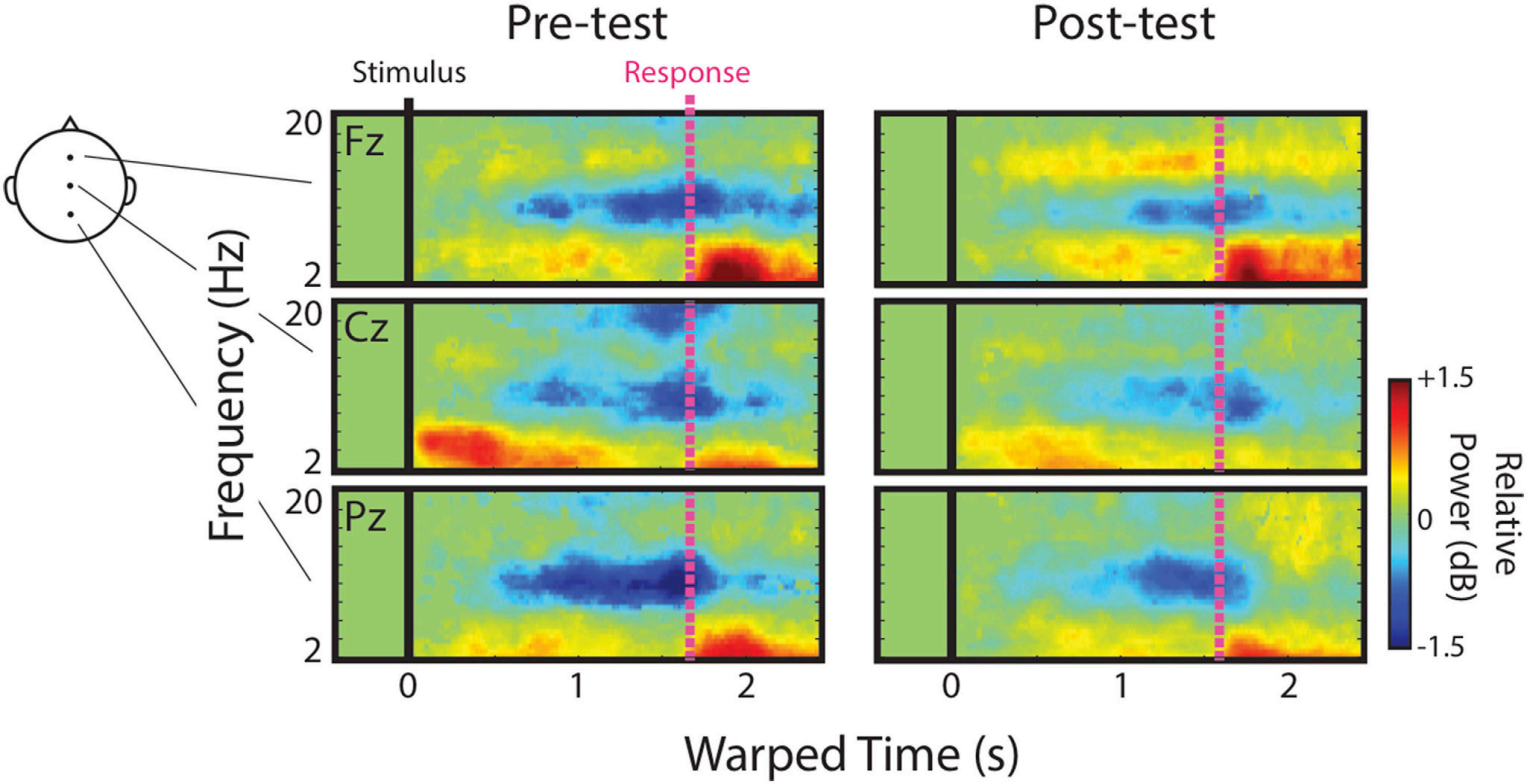
**Channel ERSPs**. ERSPs at channels Fz, Cz, and Pz at pre- and post-test.

We first describe qualitatively the ERSPs at channels Fz, Cz, and Pz before presenting detailed analyses of IC process clusters derived from ICA decomposition of channel data. Figure 3 shows mean ERSPs (averaged across participants) at each of these channels for the pre- and post-test. For clarity, ERSPs represent data averaged across speech categories (English, Bengali, and backwards), and distance (near and far). Differences across these factors were either not apparent, or appear as quantitative differences in similar ERS/ERD patterns discussed below. In these images, red indicates an increase in power relative to baseline (ERS), green indicates no change, and blue indicates a decrease in power (ERD). Because images reflect time-warped ERSPs, the relative power before mean RT (vertical pink lines) indicates activity occurring prior to key presses.

Note that the event-related dynamics of frequency bands vary across channels. For instance, the transient theta ERS (~4–8 Hz) observed during pre-test recordings, possibly in part related to components of the AEP, is strongest at Cz. Similarly, there appears to be a band of low-beta (~13–16 Hz) ERS that is present at Fz, but absent in the more posterior channels. Alpha ERD (blue portion of ERSPs near 10 Hz) is clearly present at Fz, Cz, and Pz.

In regards to possible correlates of learning, channel data provide some evidence in support of our initial hypotheses and some evidence to the contrary. Based on the original study in which accurately judged Bengali speech showed the greatest upper-alpha/low-beta ERS (Wisniewski et al., 2012), we hypothesized that this feature would increase as a result of learning. Visual comparison of low-beta ERS at Fz between pre- and post-tests seems to suggest that this was the case. We hypothesized that alpha ERD would be enhanced after learning since accurately judged English speech previously showed the greatest alpha ERD in a left temporal IC process cluster (Wisniewski et al., 2012). Channel data actually suggest the opposite. It also looks as though transient theta ERS fades from pre- to post-test, consistent with our hypothesis that this response should decrease with learning.

ERSPs derived from channel data should be interpreted with caution. One alternative explanation of increased low-beta power is that brain sources contributing to alpha ERD, possibly more so in the pre-test, are masking ERS in the low-beta band. In this case, masking release resulting from reduced alpha ERD might appear as increases in low-beta power, even if low-beta power remains stable. Additionally, several cortical sources generate theta, alpha, and beta rhythms (for review, see Buzsáki, 2006), making it difficult to relate channel data to the cortical networks generating these rhythms, some of which were specific to our hypotheses. We therefore focused primarily on analyses of ERSPs derived from IC processes.

### Electrophysiology - IC process clusters

**Figure 4:**
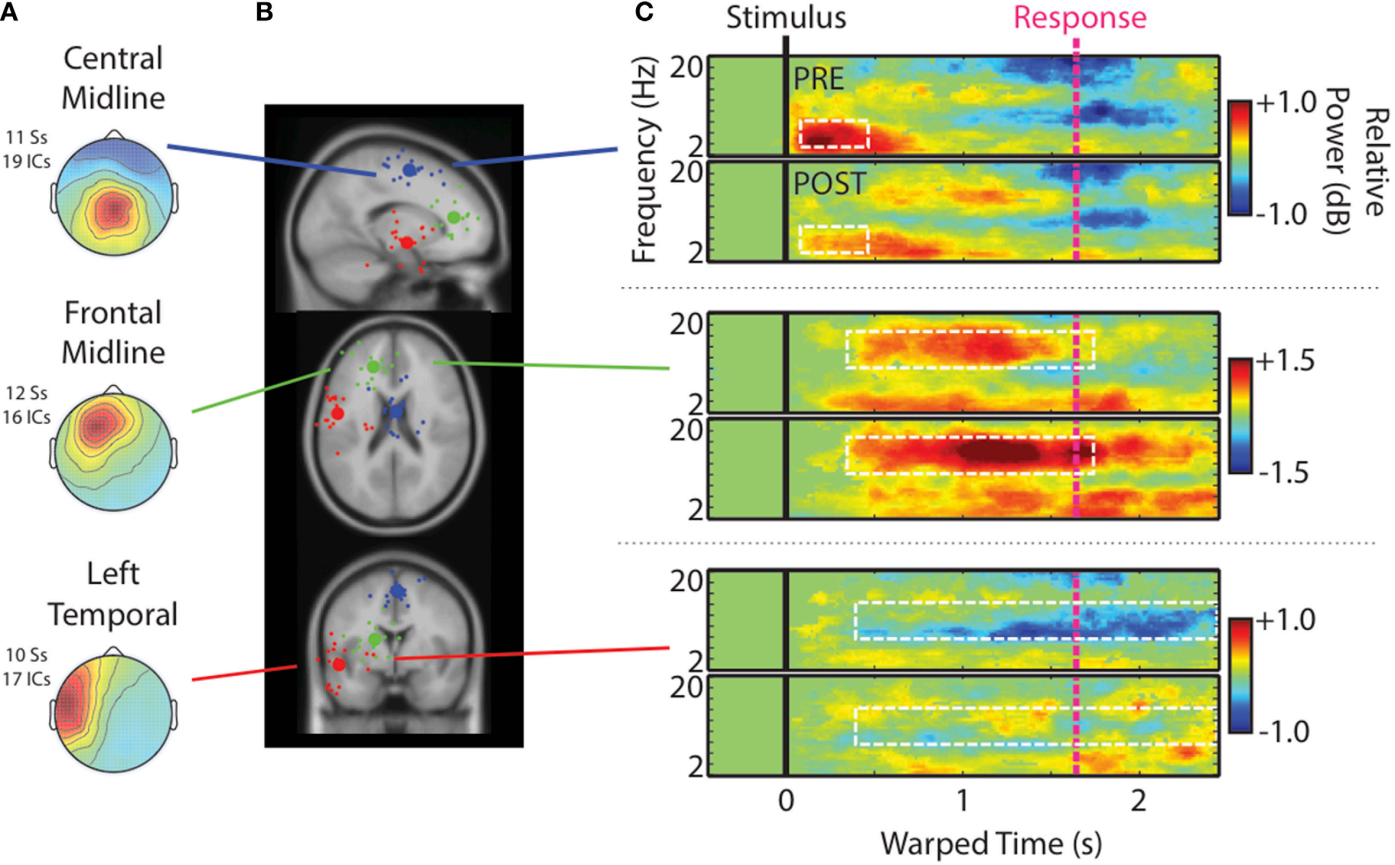
**Cluster characteristics**. **(A)** Scalp maps, **(B)** centroids of IC process clusters, and **(C)** time-warped ERSPs averaged across speech categories and participants. In **(B)**, the blue, green, and red spheres represent Central Midline, Anterior Midline, and Left Temporal clusters respectively. Rows of **(C)** show ERSPs for these different clusters. In **(C)**, the top panels show ERSPs for the pre-test and the bottom panels show ERSPs for the post-test. The white boxes indicate time-frequency windows drawn from our previous study (Wisniewski et al., 2012) and designated as regions of interest in the current analysis. These windows are: Central Midline cluster (0.15–0.6 s; 4–8 Hz), Frontal Midline cluster (0.4–1.7 s; 10–16 Hz), and Left Temporal cluster (0.5–2.45 s; 8–12 Hz).

Figure 4A shows scalp maps of IC process clusters of interest. Central midline, Frontal Midline, and Left Temporal clusters of IC processes were identified. Scalp projections of these clusters were similar to those previously observed (Wisniewski et al., 2012). Cluster centroids (large spheres) and best-fit equivalent current dipoles for each IC (small spheres) are shown in Figure 4B. Centroids were located near posterior portions of the medial frontal gyrus (Central Midline cluster; blue sphere), the left anterior cingulate cortex (Frontal Midline cluster; green sphere), and left superior temporal gyrus (Left Temporal cluster; red sphere)^5^. The absence of an individualized head model, varying numbers of electrodes between participants, and differences in the co-registration of electrode locations can greatly increase estimation error in dipole locations (Akalin Acar and Makeig, 2013). Additionally, the Central Midline cluster shows a scalp map similar to late components of AEPs, which are partly generated by sources in the temporal lobes (e.g., Debener et al., 2008). To avoid undue specification of anatomical regions, we refer to these clusters by their scalp distribution. Also, source estimates within medial cortical areas may be more susceptible to errors in lateralization due to their proximity to the boundary between hemispheres. Thus, we refrained from making any claims regarding lateralization in midline clusters.

Figure 4C shows time-warped ERSPs averaged across speech categories for the pre- (top) and post-tests (bottom) for each IC process cluster^6^. Dashed white boxes outline time-frequency windows designated for analysis (see Materials and Methods). The Central Midline cluster shows transient ERS occurring shortly after stimulus onsets between 2 and 10 Hz, but occurring most strongly in the theta range between 4 and 8 Hz. That this cluster shows a strong projection to central scalp locations and an ERSP feature similar to that seen at Cz, suggests that these ICs at least partially contribute to transient theta seen in ERSPs at channels. Note also that transient theta ERS decreases from pre- to post-test as it does in channel data.

For the Frontal Midline cluster, there is clear sustained ERS in the upper-alpha/low-beta range (10–16 Hz), appearing mostly between the stimulus (black vertical line) and response (pink vertical line). There is also an accompanying ERS in the theta range (cf., Onton et al., 2005). High-alpha/low-beta appears to increase from pre- to post-test as seen in ERSPs at Fz. Cluster ERSPs suggest that there was some masking of sustained ERS in channel data by alpha ERD. That is, sustained ERS in the cluster ERSPs appears within a wider frequency range that extends into alpha (10–16 Hz). However, the low-beta power increase from pre- to post-test seen in channels is not merely a cause of decreased alpha-masking, as it appears in the cluster ERSPs with little or no alpha ERD.

For the Left Temporal IC process cluster there were decreases in alpha ERD from pre- to post-test. There may have been some changes from pre- to post-test in theta and low-beta bands for the Left Temporal cluster, but these time-frequency windows did not satisfy our analysis criterion, and thus are not reported on. For the same reason we also do not further analyze some ERSP features of other clusters (e.g., theta in the Frontal Midline cluster).

**Figure 5:**
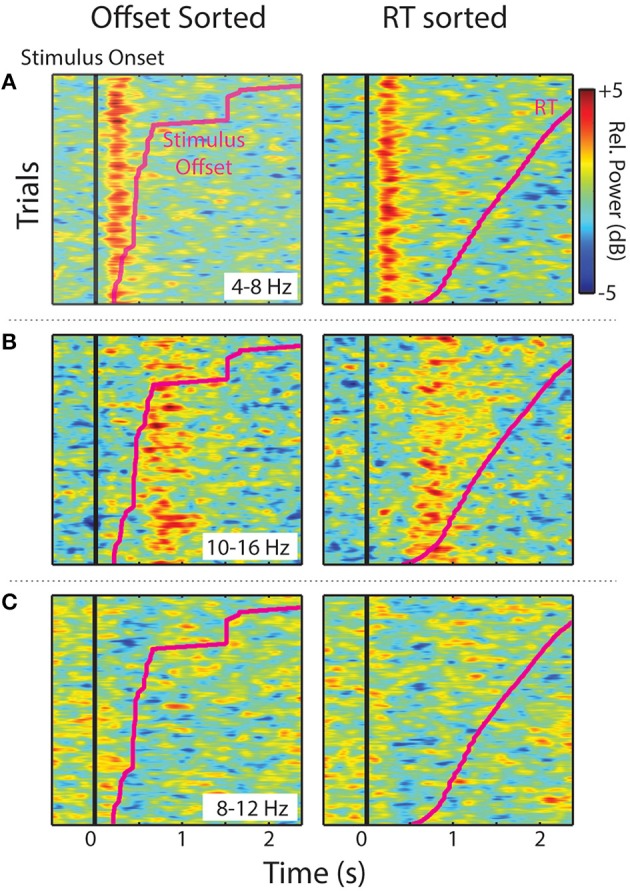
**Sorted single-trials**. Stimulus offset and RT-sorted power within specified frequency ranges for IC process clusters (all trials for all participants). Images show relative power smoothed across a moving-average 80-trial window for **(A)** Central Midline theta (4–8 Hz), **(B)** Frontal Midline upper-alpha/low-beta (10–16 Hz), and **(C)** Left Temporal alpha (8–12 Hz).

Single trials (all experimental trials for each IC, in each cluster, and smoothed over trials) sorted by stimulus offset and RT are shown in Figure 5. The two midline IC process clusters showed that ERS in the theta (Central Midline) and upper-alpha/low-beta ranges (Frontal Midline) was clearly time-locked to stimulus onsets. Increases in power were aligned vertically instead of diagonally like the individual trial stimulus offsets and RTs (pink lines). This suggests that observed ERS is not related to sound offset or response planning/preparation processes. It is also important to note that the Frontal Midline cluster shows sustained upper-alpha/low-beta ERS in single trials and that this ERS sustains longer in trials with longer RTs. That is, longer RT trials show ERS up to ~1.8 s in the RT sorted plot, whereas short RTs generally show little ERS past ~1 s. Single-trial sorted alpha power for the Left Temporal cluster is less clearly aligned to stimulus onsets. However, there does appear to be some evidence of vertical alignment of alpha ERD around 0.4–1 s.

**Figure 6:**
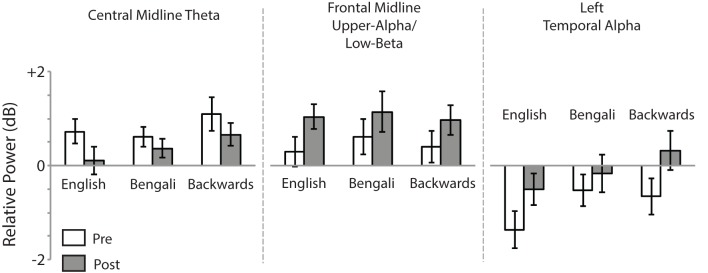
**Mean relative power**. Relative power within time-frequency windows of interest for the Central Midline, Frontal Midline, and Left Temporal IC process clusters. Error bars show standard errors of means.

Figure 6 shows mean relative power for each speech category within the time-frequency windows of interest. For the Central Midline cluster there was a main effect of Test [*F*_(1, 18)_ = 6.23, *p* = 0.022, η^2^_*p*_ = 0.26], demonstrating a decrease in theta ERS from pre- to post-test. The main effect of Speech Category was also significant [*F*_(2, 36)_ = 3.73, *p* = 0.034, η^2^_*p*_ = 0.17]. In our previous study we observed larger transient ERS for backwards speech in a similar cluster, which appears to be replicated in the post-test here. *Post-hoc* paired comparisons (FDR corrected) found that the backwards speech category induced marginally significant greater theta ERS than English [*t*_(18)_ = 2.21, *p* = 0.060, *r*^2^ = 0.21] and Bengali speech [*t*_(18)_ = 2.60, *p* = 0.054, *r*^2^ = 0.27]. The difference between English and Bengali speech was not significant (*p* > 0.65). The Speech Category x Test interaction was not significant (*p* > 0.45).

For the Frontal Midline cluster's upper-alpha/low-beta, there was a significant main effect of Test [*F*_(1, 15)_ = 6.97, *p* = 0.019, η^2^_*p*_ = 0.032], showing that ERS in the upper-alpha/low-beta range increased from pre- to post-test. The main effect of Speech Category and Speech Category × Test interaction were not significant (*p* > 0.40).

For the Left Temporal cluster there was a main effect of Test [*F*_(1, 16)_ = 12.75, *p* = 0.003, η^2^_*p*_ = 0.44], indicating that alpha ERD decreased from pre- to post-test. There was also a significant effect of Speech Category [*F*_(2, 32)_ = 8.66, *p* = 0.001, η^2^_*p*_ = 0.35], relating to our previous report of the largest ERD having been for English speech. Indeed, *post-hoc* paired *t*-tests revealed that English speech induced greater alpha ERD than Bengali [*t*_(16)_ = 3.48, *p* = 0.009, *r*^2^ = 0.43] and backwards speech [*t*_(16)_ = 3.03, *p* = 0.012, *r*^2^ = 0.36]. The Speech Category x Test interaction was only marginally significant [*F*_(2, 32)_ = 3.11, *p* = 0.058, η^2^_*p*_ = 0.16].

Analysis of Near vs. Far stimuli revealed only main effects of Test for each 2 (Test) × 2 (Distance) ANOVA, replicating those above (*p* < 0.05). No significant main effects of Distance or Distance × Test interactions were found for any IC process cluster (*p* > 0.15).

### Electrophysiology - AEPs

As noted above, the Central Midline cluster shows a scalp map very similar to that of AEP components N1 and P2. Using ICA to decompose high-density EEG recordings from an auditory oddball task, Debener et al. (2005) observed a similar central midline cluster of IC processes that showed N1, P2, and an additional P3 component. The transient theta ERS observed here could be related to any one or all of these features.

**Figure 7:**
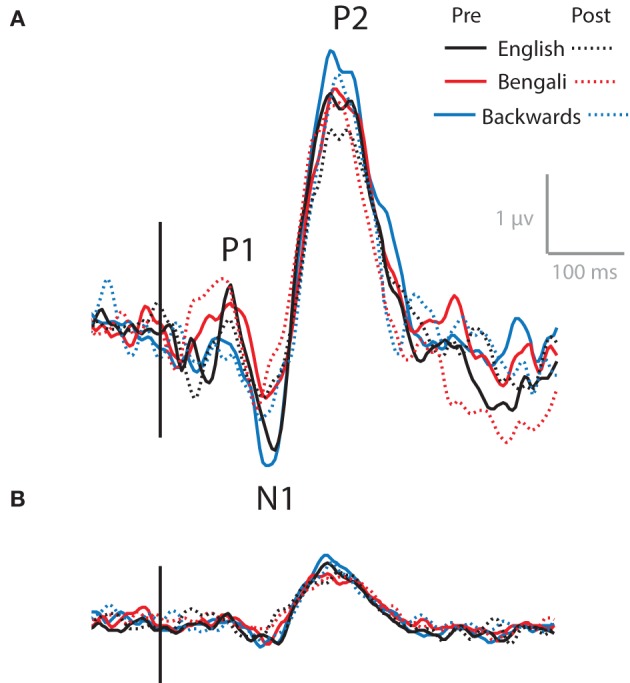
**AEPs**. AEPs for data backprojected to channels surrounding Cz from all non-artifactual ICs **(A)** and ICs within the Central Midline IC process cluster **(B)**.

Figure 7 shows the ERP at channels surrounding Cz, combining all non-artifactual sources in backprojection (Figure 7A). These AEPs represent typical waveforms after removing eye- and movement-related artifacts form the data. ERPs produced after backprojecting only ICs in the Central Midline cluster are also shown (Figure 7B). Waveforms show N1 and P2 peaks for both backprojections. P1 appears in the data after backprojecting all non-artifactual sources, but is less apparent in the data backprojecting only ICs within the Central Medial cluster.

As with transient theta ERS, it appears as though N1 and P2 decrease from pre- to post-test, with the largest amplitudes for backwards speech. That is, the solid lines show larger peaks than the dashed lines, and the blue lines (backwards speech) generally show greater amplitudes than the red (Bengali) and black (English) lines. This is evident in both backprojections. However, no significant effects were found in 3 (Speech Category) × 2 (Test) ANOVAs for N1 or P2 components for waveforms obtained using Central Midline cluster ICs (*p* > 0.10). Also, note that these time-domain features do not extend into the full range of ERS seen in ERSPs (up to ~600 ms) and likely do not fully account for ERS seen in the Central Midline cluster (Makeig et al., 2004).

### Relationship of EEG to behavior

**Figure 8:**
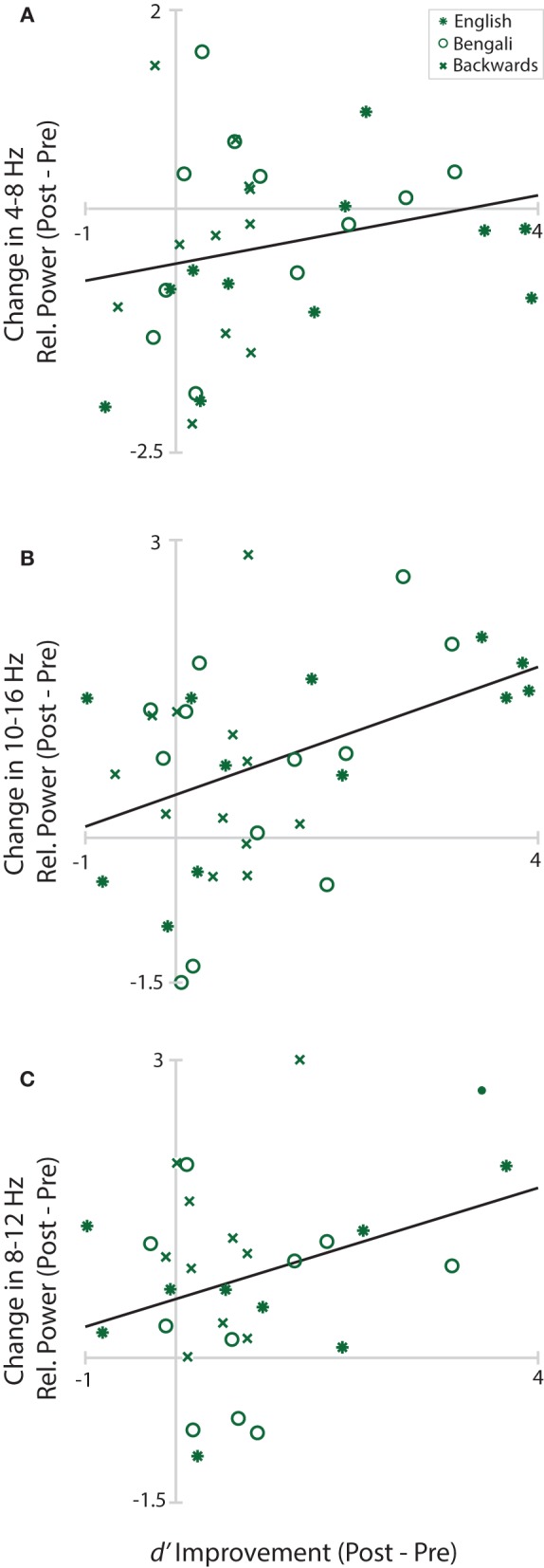
**Learning/EEG correlations**. Scatterplots of improvement scores and changes in relative power from pre- to post-test in each cluster and time-frequency window. Asterisks mark English, circles mark Bengali, and Xs mark backwards speech.

Figure 8 shows changes in Central Midline theta, Anterior Midline high-alpha/low-beta, and Left Temporal alpha plotted as a function of *d*′ improvement scores (Post-Pre-test *d*′). Solid black lines in the figure depict linear fits. Positive values on the y-axis indicate greater power within the designated time-frequency window in the post-test. Positive values on the x-axis depict improvements in perceptual sensitivity to distance. The only significant relationship was between changes in Frontal Midline cluster high-alpha/low-beta and improvement scores, *r*_(34)_ = 0.42, *p* = 0.033. The relationship was positive, trending such that greater increases in relative power from pre- to post-test were associated with greater improvements in distance judgments. Neither the correlation of improvement scores with Central Midline theta, *r*_(31)_ = 0.22, *p* = 0.221, nor Left Temporal alpha, *r*_(28)_ = 0.34, *p* = 0.104, reached significance.

## Discussion

In the current study, we examined how perceptual training with speech sounds differing in familiarity altered distance perception accuracy and event-related spectral dynamics of EEG. An ICA approach to EEG analysis was used to characterize how independent and distributed brain processes relate to variations in distance perception accuracy. In part, the study served to determine whether or not EEG features shown to correlate with speech familiarity effects (Wisniewski et al., 2012) relate to within-experiment learning effects on distance perception. It was also intended to extend neuroimaging studies of human auditory distance perception beyond investigations of representational differences for near and far sounds in canonical auditory processing regions of cortex (cf., Mathiak et al., 2003; Kopčo et al., 2012; Altmann et al., 2013). We hoped to characterize how the cortical dynamics involved in active listening for distance cues changed with training.

Training led to more accurate distance perception across English, Bengali, and backwards speech categories, with greater improvement for familiar speech sounds (i.e., forwards speech). Replicating previous EEG work (Wisniewski et al., 2012), speech familiarity was related to differences in spectral perturbation patterns in Central Midline and Left Temporal clusters of IC processes. In the Central Midline cluster, backwards speech appeared to lead to the greatest transient theta ERS. English led to the greatest alpha ERD in a Left Temporal cluster. Perceptual learning in all speech categories was associated with a reduction in both of these cortical responses. In contrast, sustained upper-alpha/low-beta ERS localized at or near anterior regions of the medial frontal cortex was amplified after training. Furthermore, increases in this sustained ERS were positively correlated with learning.

The advantage of forward over backwards speech in terms of auditory distance perception has been previously reported (McGregor et al., 1985; Brungart and Scott, 2001; Wisniewski et al., 2012), but the present data seems to be the first evidence for a learning advantage. This advantage cannot be explained based on general auditory processing enhancements (e.g., Voss et al., 2004; Kolarik et al., 2013), because the different speech categories contained comparable known acoustic cues to distance. Furthermore, if performance differences were driven by global increases in auditory sensitivity, the dynamics of the left temporal IC cluster should have been most clearly related to changes in performance given its nearness to traditional auditory processing regions (Recanzone et al., 1993; Weinberger, 2007). Left temporal alpha ERD actually decreased from pre- to post-test, suggesting decreased involvement of this region after training. A similar trend was qualitatively observable in AEPs, which decreased in amplitude rather than increased as is typically associated with auditory perceptual learning (e.g., Orduña et al., 2012).

The advantage of learning associated with forward speech might actually reflect a disadvantage for learning backwards speech. For instance, listeners' initial difficulty judging differences between near and far backwards speech might have interfered in some way with their ability to benefit from training. Learning does tend to be limited for stimulus contrasts that are difficult to discriminate before training in comparison to contrasts that are easier (e.g., Lawrence, 1952; Orduña et al., 2012; Church et al., 2013). However, in past studies difficulty has typically been manipulated by modifying physical stimulus differences. Although acoustic features within speech sounds were not identical across speech categories, available cues to distance were highly similar (see Figure 1). Differences in learning, even if related to pre-training difficulty, thus are more likely to reflect differences in processing inherent to judging forward vs. backwards speech sounds^7^.

Why is it then that listeners were better able to learn to distinguish the distances of sound sources producing forward speech? EEG data provide some clues. First, greater transient theta ERS was predictive of poor auditory distance perception performance in both the current and previous study. Specifically, relatively large transient ERS was associated with less accurate perception of backwards speech sounds, and decreases in this transient response accompanied increases in performance from pre- to post-test. One possibility is that transient ERS may be a sign of processing that is irrelevant or counterproductive for performing the auditory distance judgment task. Several ERP studies of auditory distraction have shown that novelty and orienting responses, such as MMN (e.g., Schröger, 1996) and P3 (e.g., Berti, 2013) components, are associated with decreases in performance on some primary perceptual task. For instance, even though participants may be instructed to ignore a task-irrelevant auditory stream, oddball sounds within that stream lead to both an increase in RT for a primary visual task, and a larger amplitude P3 in the ERP time-locked to auditory events (Berti, 2013). Other work analyzing time-frequency features of EEG have associated transient theta ERS to novelty/orienting responses in similar oddball paradigms, and have suggested that such responses reflect obligatory “attention switching” caused by obtrusive sensory events (Dietl et al., 1999; Barry et al., 2012). The transient ERS seen here may be related to these types of obligatory processes, especially for unfamiliar and unnatural sounding backwards speech, making it harder for listeners to execute the primary task of determining distance from relevant acoustic cues. A decrease in such novelty-driven interference occurring after multiple stimulus presentations (i.e., habituation; Friedman et al., 2001) may make it easier for participants to devote resources to the task at hand (Schröger, 1996; Berti, 2013). This interpretation makes the yet to be tested prediction that individuals with extensive experience localizing backward speech should perform as well at localizing backwards speech as forward speech, and should show comparable cortical activation patterns for either stimulus type.

While transient theta ERS decreased after training, sustained upper-alpha/low-beta ERS attributed to the medial frontal cortex increased. In one study analyzing the spectral dynamics of a similar frontal midline cluster of IC processes, both sustained theta and low-beta power increased as more items were held in working memory (Onton et al., 2005). There are also several fMRI and PET studies of auditory attention that show greater activation in prefrontal and anterior cingulate areas in tasks requiring increased attentional (Zatorre et al., 1999; Benedict et al., 2002; Janata et al., 2002; Mulet et al., 2007; Ahveninen et al., 2013; Uhlig et al., 2013) or memory resources (Zatorre et al., 1994). Others have reported increased activation in similar regions when specific acoustic features need to be tracked over time (Janata et al., 2002; Uhlig et al., 2013). Sustained upper-alpha/low-beta ERS may similarly relate to higher-level processing important for either sustained attention-related effects on auditory perception or working-memory related processes important for the integration, extraction, and/or retention of multiple acoustic cues to distance. Learning may involve increased engagement of these networks during listening.

We cannot provide a clear answer as to why sustained ERS features increase in parallel to decreases in transient ERS. Although a reduction in orienting/novelty processing might make it easier to sustain task-related processing in other regions, it is also possible that the relationship is reversed. For instance, some data suggests that increasing working memory demands can decrease ERP signatures of involuntary orienting to distracting sounds (Lv et al., 2010). In this vein, EEG signatures of orienting may be reduced because listeners are engaging more in sustained processing. Another possibility is that it takes training over several trials for listeners to reliably use appropriate listening strategies and that there is no causal relationship between the observed transient and sustained responses. Rather, there is only a transition in processing because listeners discovered that a sustained attentional or memory related strategy was effective. Regardless, in this study activity in frontal cortical networks seem to be more closely related to performance and learning than cortical networks ostensibly viewed as “auditory processing” regions.

### Psychophysiological investigations into auditory learning

By far, most psychophysiological studies of human auditory learning have employed evoked-potential methods. For instance, there exist several studies reporting that N1, P2, and MMN components of the AEP are plastic, showing changes in amplitude and latency with learning (e.g., Tremblay et al., 2001; Atienza et al., 2002; Gottselig et al., 2004; Boaz et al., 2010; Orduña et al., 2012). Learning-related modifications to these evoked responses are generally observed less than 500 ms post-stimulus onset. In contrast, we found that the strongest correlate of learning was induced ERS in an upper-alpha/low-beta frequency band. The presence of this ERS sustained well past typical evoked-potential latencies (~1700 ms post-stimulus onset). Familiarity with English speech was also associated with a sustained EEG feature. Namely, greater alpha ERD at time points exceeding 500 ms. These features would go undetected in a typical ERP study of auditory learning.

It is common to observe sustained ERS and ERD features during listening. Pesonen et al. (2006) asked listeners to indicate whether or not a spoken probe word was presented in a previous set of spoken words. Not only did the probe induce alpha ERD from 400 to ~1000 ms after probe onset, but theta and low-beta ERS was present up to ~1400 ms. Furthermore, words in the memory set did not induce low-beta ERS, suggesting that this feature was related to auditory recognition rather than encoding. In one recent study, the degree of alpha ERD corresponded with perception of a tone as high or low, even when the physical stimuli accompanying these perceptions were identical (Hartmann et al., 2012). Others have found that alpha ERD precedes the presentation of informative auditory stimuli, suggesting a relationship between oscillatory activity and anticipatory attention (e.g., Bastiaansen and Brunia, 2001). These studies are only a sample of demonstrated long-duration event-related modulations of the EEG spectrum during listening tasks (for review, see Krause, 2006; Weisz et al., 2011).

The evoked-potential approach to studying auditory learning assumes that non-phase locked spectral perturbations in EEG are noise, and that learning is mostly related to changes in evoked activity that occur close in time to stimulus onset. Although evoked–potential changes likely play an important role in auditory learning, these measures may fail to capture many learning processes. Because oscillatory dynamics of EEG seem to be related to auditory memory (Pesonen et al., 2006), subjective impressions of physically identical sounds (Hartmann et al., 2012), and active listening (Bastiaansen and Brunia, 2001), it seems likely that their examination could be informative in understanding how training leads to changes in perceptual acuity. The data reported here show that sustained phase-independent EEG features do change with learning. Future auditory learning studies may benefit from consideration of how both evoked-potential and oscillatory dynamics of EEG relate to learning-induced cortical plasticity.

### Further considerations and caveats

Both the current and our earlier study represent initial attempts to characterize neural correlates of auditory distance perception in the oscillatory dynamics of EEG attributed to a distributed network of brain regions. Previous neuroimaging research has focused mainly on responses attributed to temporal brain regions (Mathiak et al., 2003; Kopčo et al., 2012; Altmann et al., 2013). Given the absence of data to support strong hypotheses regarding the activity of other cortical circuits that might contribute to auditory distance perception, a data-driven analysis approach was taken. We first identified clusters of IC processes that were related to performance and that showed clear event-related spectral dynamics (Wisniewski et al., 2012). Expanding on that original work, oscillatory dynamics of those processes were examined before and after training. Although portions of the data are consistent with prior work (i.e., temporal clusters of ICs show task-related dynamics; Mathiak et al., 2003; Kopčo et al., 2012; Altmann et al., 2013), previously ignored non-auditory cortical networks were found to relate most clearly to learning-related improvements in distance perception. Future hypothesis-driven studies are needed to validate the effects and interpretations presented here. Our identification of a distributed cortical network involved in auditory distance perception and learning should facilitate the development of such experiments.

Our work does not directly support several previous proposals regarding how learning impacts distance perception, but these proposals should not be dismissed. It remains possible that more subtle modifications to perceptual processing (e.g., Voss et al., 2004; Kolarik et al., 2013) indexed by higher-frequency spectral dynamics (Ahveninen et al., 2013), phase-locked responses (Orduña et al., 2012), or receptive fields of single neurons (Weinberger, 2007), contribute to performance improvements. Similarly, speech vs. non-speech representational differences in the brain may be related to performance, and detectable with other neuroimaging methodologies that are better suited for exploring neural processing with finer spatial resolution.

We collected no data verifying that listeners perceived stimuli as differing along a spatial dimension. That is, even though listeners discriminated far from near sounds, they may have perceived them as varying along some other dimension (e.g., background noisiness or timbre). Our intensity-normalized sounds also differ from most natural situations in which intensity differences are highly salient indicators of source distance (Coleman, 1963). This likely reduced the degree to which our stimulus set sounded natural. However, given that sounds contained viable cues to distance and that participants picked up on these cues (i.e., they performed at above chance levels), it seems likely that the sounds were perceived as varying in distance. Furthermore, the data show that listeners utilized distance cues, and learned about them, regardless of whether or not they truly perceived sounds as coming from sources at near or far locations.

As a final caveat, we have not compared processing during performance of the auditory distance perception task to processing during other auditory discrimination or spatial judgment tasks. The findings reported here may not be specific to distance perception. In fact, evidence that the strongest EEG correlates of performance are in non-auditory regions with spectral dynamics similar to those observed by others in non-auditory tasks would suggest that they are not. We do not see this as a weakness of the study, but rather a departure from previous approaches that serves to more fully characterize human brain dynamics during listening and distance judgment. One might also be concerned by the lack of clear differences in cortical activity induced by the processing of near and far sounds, given several studies suggesting that near and far distances are represented differently in the brain (e.g., Mathiak et al., 2003; Kopčo et al., 2012; Altmann et al., 2013). The EEG dynamics reported here are correlated with accuracy in distance perception even though they are not correlated with the dimension of distance. Our particular methodology may have either been insensitive to the detection of differences between near and far, or there exist large differences between individuals in regards to how they deal with this level of detail, making it difficult to detect differences in averaged data (cf. Wisniewski et al., 2014).

## Conclusions

In two studies we have found task-related EEG oscillatory dynamics attributed to sources at or near both auditory and non-auditory brain regions. The earliest published neuroimaging work on human auditory distance perception suggested involvement of a distributed network of brain processes (Seifritz et al., 2002). However, most of the following work did not analyze activity in non-auditory brain regions (Mathiak et al., 2003; Kopčo et al., 2012; Altmann et al., 2013), instead restricting analyses to regions of interest in temporal cortex. The clearest conclusion that comes out of our studies is that activity in non-auditory cortical networks is associated with, and likely contributes to, auditory distance perception accuracy. These networks may be particularly important when effects on perception cannot be accounted for by the presence, absence, or manipulation of acoustic cues to distance. Given that we observed learning-related modifications to sustained ERS/ERD features, auditory perceptual learning research may benefit from explorations into how these non-phase dependent EEG dynamics relate to learning. Future work in both auditory distance perception and learning may find it useful to look beyond AEPs, which capture only a portion of the event-related processes observable in EEG (Makeig et al., 2004).

## Author contributions

Conceived and designed the experiment: Eduardo Mercado III, Barbara A. Church, Matthew G. Wisniewski. Performed the experiment: Matthew G. Wisniewski, Klaus Gramann. Analyzed the data: Matthew G. Wisniewski. Contributed reagents/materials/analysis tools: Scott Makeig, Klaus Gramann. Wrote the paper: Matthew G. Wisniewski.

### Conflict of interest statement

The authors declare that the research was conducted in the absence of any commercial or financial relationships that could be construed as a potential conflict of interest.

## Footnotes

^1^ A subset of environmental sounds were recorded and also tested, but data associated with these sounds are not discussed due to drastic acoustic differences between these environmental sounds and speech.

^2^ A One-Way ANOVA on stimulus durations showed no significant effect of Speech Category (*p* > 0.70).

^3^ An FDR procedure introduced by Benjamini and Hochberg (1995) was used wherein the false discovery rate for hypothesis *k* is bounded by $\frac{np\left(k\right)}{k}\leq 0.05$. Here, *n* is the number of tests, *k* denotes the rank of the p value being corrected (from small to large), and *p(k)* is the *k*-th smallest of the *p* values. In the results, *p* values are reported as corrected by the left side of the equation. FDR was applied separately for each family of post-hoc comparisons. For example, paired comparisons on data from one cluster of ICs were treated as a family separate from other clusters.

^4^ The *c* signal detection parameter was calculated using Hit and False Alarm rates across all trials to determine if there was a bias to respond near or far. There existed a slight bias to respond “Near” (*M* = −0.20, *SE* = 0.06) that was significant [*t*(14) = 3.50, *p* = 0.004, *r*^2^ = 0.47]. Bias did not change from pre- to post-test, *t* < 1.

^5^ Locations listed in the text refer to the gray matter nearest to cluster centroids.

^6^ Unmasked and non-time-warped ERSPs are shown in Supplementary Material.

^7^ An alternative hypothesis is that audiospatial learning for speech and non-speech stimuli involve different mechanisms (Loebach and Pisoni, 2008), but the current data provide no support for this possibility. Rather, differences in EEG between speech categories appear to reflect quantitative differences in cortical activity. Another possibility is that backwards speech is processed differently because of its more graded onsets (He, 2001). However, distance cues within the speech sounds, such as SNR-related differences between near and far stimuli, were present throughout the duration of each stimulus. Furthermore, explicit training produces comparable perceptual learning curves for features occurring at sound onsets and offsets (Mossbridge et al., 2006, 2008), suggesting that the presence or absence of particular time domain features within speech sounds is unlikely to strongly constrain an individual's ability to learn to differentiate auditory distance cues. Although there are acoustic differences between forward and backwards speech, all known acoustic distance cues are comparable between these two categories. If other acoustic features that differ between forward and backwards speech are useful for distance perception, they have yet to be tested experimentally. However, if they exist, they could in principle affect the ease with which one can learn.
