# Supplementary material for: Brain dynamics that correlate with effects of learning on auditory distance perception
Source: Front Neurosci. 2014 Dec 9;8:396. doi: 10.3389/fnins.2014.00396 (PMC4260497; doi:10.3389/fnins.2014.00396)
Supplement: Supplementary file 1 [file DataSheet1.DOCX]

**Appendix A**

This appendix describes additional details regarding time-frequency analyses and clustering procedures employed in the study.

*Single-trial Relative Power Sorting:* Response times are variable. A response-related desynchronization (see Pfurtscheller & Lopes da Silva, 1999) or synchronization (Delorme et al., 2007) in EEG may occur up to seconds preceding actual response onsets. Although time-warped ERSPs can partially disambiguate stimulus-related and response-related time-frequency features in EEG by distinguishing features occurring before and after response onsets, some ERS and ERD patterns occurring before the response may still be associated with response aspects of the task. Similarly, as stimulus durations varied in the current stimulus set, responses associated with stimulus offsets could contribute to ERS/ERD features in time-warped ERSPs.

Here, single-trial sorting of relative power within a frequency region of interest served to further disambiguate stimulus, stimulus offset, and response-related EEG features. Like with the time-warped ERSPs, each IC’s single-trial event-related spectrum was computed using Morlet wavelets (2 cycles at the lowest frequency to 10 cycles at the highest; 2-20 Hz) in 200 overlapping windows. Absolute power within the frequency band of interest for each trial was normalized by dividing by an IC’s mean power within that band across all time points and trials. The result of this process for a single-trial was a one-dimensional vector representing relative power for a single frequency band at 200 time points. Vectors corresponding to all trials and ICs were sorted by stimulus offset or RT, giving a matrix with a size of trials x time points. Smoothing over trials was accomplished by using a moving average 80-trial window (smoothing over rows of the matrix). The result was a single image, showing how ERS or ERD within a frequency band of interest related to response latencies (stimulus onsets at time 0, response onsets at varying latencies). Given a stimulus-related ERS or ERD pattern, the image should show alignment of relative power changes to the onset of the stimulus rather than the response. Given an offset or response-related pattern, relative power changes should align diagonally, showing increasing latency with increasing offset/response times.

*IC process clustering:* K-means was used to cluster ICs with similar scalp map topographies, equivalent current dipole locations, ERSPs, and mean log power spectra. Except for dipole location, each of these measures was compressed using principle components analysis (PCA) into a 10 dimensional vector for each IC. Dipole locations were 3-dimensional. To compensate, dipole information was weighted by a factor of 10 in K-means clustering. ERSPs were weighted by a factor of 3 and the other measures were weighted by 1. ICs with mean characteristics at least 2.5 standard deviations from any cluster’s centroid were rejected from further analyses as outliers. Clusters of ICs identified in the current study were generally similar to those reported previously (Wisniewski et al., 2012), except for being slightly smaller. Differences were a result of using data from both pre- and post-tests to calculate clusters, and altered criteria for selecting clusters (e.g., greater weighting of ERSPs and a lower standard deviation used to identify outliers).

Note that in our previous study a right-lateralized parietal cluster was found to show greater ERD in an EEG band within the alpha range for backwards speech (Wisniewski et al., 2012). However, the new clustering led to differences in the ICs assigned to a similar right parietal cluster. For instance, ICs previously assigned to central parietal and right temporal clusters ended up in a right lateralized parietal cluster and vice versa. This difference in assignment led to differences in the ERSPs and greater ambiguity in relation to location. Therefore, this cluster is not reported in the text. However, exploratory analyses using a 3 (speech category) x 2 (test) repeated measures ANOVA on ERSPs of the new right parietal cluster found no significant effects [*p*>.3].

**Appendix B**

**
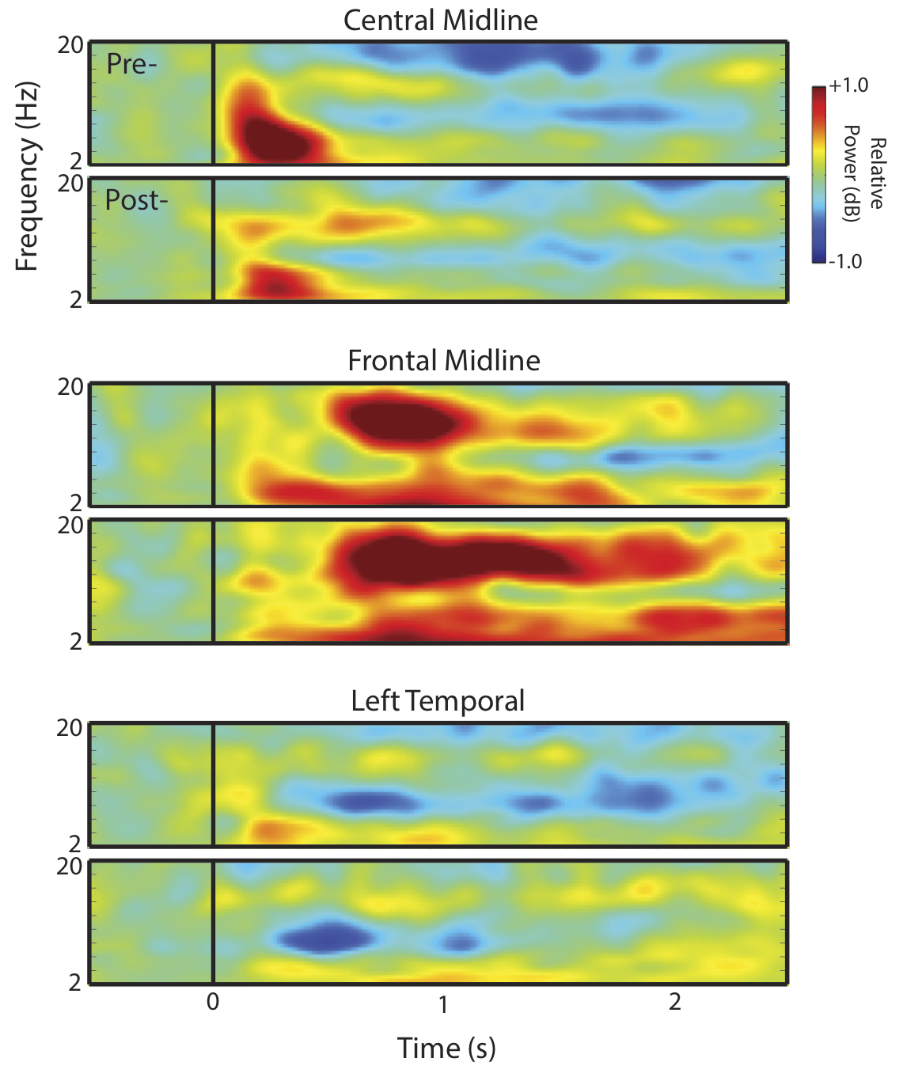
**

*Figure B1:* Unmasked and non-time-warped ERSPs (averaged across all ICs and speech categories) for each cluster in the pre- and post-tests. Note that spectral dynamics reported in the paper exist in the unmasked ERSPs.
